# A Study on Effect of Electroacupuncture on Gene Expression in Hypothalamus of Rats with Stress-Induced Prehypertension Based on Gene Chip Technology

**DOI:** 10.1155/2015/621237

**Published:** 2015-07-01

**Authors:** Xiaojia Xie, Yan Guo, Qingguo Liu, Zhaoyang Wang, Changqing Guo

**Affiliations:** School of Acupuncture, Moxibustion and Tuina, Beijing University of Chinese Medicine, Beijing 100029, China

## Abstract

*Objective*. To explore the effect of electroacupuncture (EA) on gene expression in the hypothalamus of rats with stress-induced prehypertension and try to reveal its biological mechanism with gene chip technology. *Methods*. The stress-induced hypertensive rat model was prepared by combining electric foot-shocks with generated noise. Molding cycle lasted for 14 days and EA intervention was applied on model + EA group during model preparation. Rat Gene 2.0 Array technology was used for the determination of gene expression profiles and the screened key genes were verified by real-time fluorescence quantitative PCR method. *Results*. Compared with the blank group, 234 genes were upregulated and 73 were downregulated in the model group. Compared with the model group, 110 genes were upregulated and 273 genes were downregulated in model + EA group. The PCR results of the key genes including HSPB1, P2RX4, PPP1R14A, and TH are consistent with that of gene chip test. *Conclusion*. EA could significantly lower blood pressure of stress-induced prehypertension rats and affect its gene expression profile in hypothalamus. Genes and their signal transduction pathway that related to the contraction of vascular smooth muscle, concentration of Ca^2+^, and excitability of sympathetic nerve may be involved in EA's antihypertensive mechanism.

## 1. Introduction

Stress-induced hypertension refers to high blood pressure that is caused by the long-term tension and stress [1]. In recent years, the prevalence of hypertension significantly increased in people who worked in high-pressure environment [2]. Present studies showed that stress-induced hypertension is of complicated pathogenesis which mainly involves the hypothalamus-pituitary-adrenal cortex (HPA) axis [3], sympathetic nerve-adrenal medulla (SAM) system [4], renin-angiotensin-aldosterone system (RAS) [5], NO/NOS system [6], and neuropeptide Y (NPY) [7].

In most conditions, the occurrence of hypertension is a chronic process. The 7th report released by the Joint National Committee on the Prevention, Detection, Evaluation, and Treatment of High Blood Pressure (JNC-7) pointed out that prehypertension is a transition stage during which the blood pressure rises from normal to diagnostic hypertension. In this stage, the systolic blood pressure ranges within 120~139 mm Hg and (or) diastolic blood pressure ranges within 80~89 mm Hg [8]. The study found that the incidence of cardiovascular events in the people with prehypertension in the future was two times as that of the people with normal blood pressure [8]. It is also proved that stress-induced hypertension is preceded by the damage of target organs like blood vessels [9], heart [10], brain [11], and kidneys [12] and changes of the expressions of relative proteins and genes. Therefore, early stage treatment could effectively reduce the incidence of high blood pressure and protect target organs. Also, studies have shown that treatment in the prehypertension stage can significantly reduce admission rate and mortality of hypertensive patients and enhance the protection of target organs of the disease [13].

Electroacupuncture therapy was widely applied in the treatment of various kinds of hypertension-related cardiovascular diseases in clinical practices. It is an effective means for the regulation of blood pressure and remission of the symptoms caused by hypertension [14, 15]. Lots of researches confirmed that acupuncture reduces blood pressure by immune system [16], nervous system [17], NO/NOS system [18], and vascular endothelial cell [19].

Gene chip technology, characterized by high sensitivity and throughput, is a new method to analyze gene expression. It could detect multiple gene expressions, and at the same time it provides a platform for the study of multiple genes influencing hypertension [20]. However, the previous researches on treatment of hypertension were concentrated more on antihypertensive treatment and protection of target organs, which are applied in the middle and late stages. Fewer researches focused on gene expression and intervention of prehypertension. The application of gene chip technology for the study of acupuncture's intervention effect on prehypertension has not been reported.

In this study we will explore acupuncture's effect on stress-induced prehypertension and try to systematically reveal its biological mechanisms from the aspect of gene by monitoring key genes and targets with gene chip technology, thereby providing a new idea for the prevention and treatment of hypertension by acupuncture.

## 2. Material and Methods

### 2.1. Animals Preparation

Specified-pathogen-free (SPF) Wistar rats were purchased from Beijing Vital River Laboratory Animal Technology Co. Ltd., with license number SCXK (Beijing) 200223. Experimental animals were raised in clean cabinets and were given free access to water and food. A controlled environment with a temperature of (20 ± 1)°C, humidity of 50%, and 12-hour light-dark cycle was maintained throughout the whole study. All procedures for animal experiments were conducted in accordance with World Health Organization's International Guiding Principles for Biomedical Research Involving Animals and were approved by the Animal Care and Use Committee at Beijing University of Chinese Medicine.

### 2.2. Grouping

Twenty-seven SPF Wistar male rats of 220 ± 30 g were randomly divided into 3 groups, namely, blank control group, model control group, and model + EA group (*n* = 9/group).

### 2.3. Model Preparation

The stress-induced hypertensive rat (SIHR) model was established by electric foot-shocks combined with generated noise. Rats in model control group and model + EA group were placed in a cage (22 cm × 22 cm × 26 cm) with a grid floor and received electric foot-shocks (30 V, 5 ms duration, 2~25 s intervals) and noises (80–100 db) produced by a buzzer (MG-2TYPE, Huai Bei Zheng Hua, China) randomly delivered by a computer. The procedure was repeated twice a day (8:00 to 10:00 in the morning and 2:00 to 4:00 in the afternoon). Rats in blank control group were put into the same cage in the same time period, with no foot-shocks or noise.

### 2.4. Intervention

All the rats in three groups were loosely immobilized in a specially made restrainer with four limbs exposed.* Taichong* (LR3) is located in the dorsum of the foot, in the depression anterior to the junction of the first and second metatarsals.* Quchi* (LI11) is located in the proximal end of radius, in the depression lateral and anterior to the elbow joint.

Model + EA group was as follows: the needles (0.32 mm ∗ 25 mm, purchased from Suzhou Acupuncture Goods Co. Ltd.) were directly inserted into LR3 and LI11 bilaterally for about 1.5–2 mm and 4 mm, respectively. After the insertion, Hans (LH202H, Beijing) LH202H was connected with Quchi and Taichong to form a circulation of 1 mA with a frequency of 2 Hz. The needles were then withdrawn after a 20 minutes' retention. The acupuncture intervention took place once a day (from 5 pm) since the first day of model preparation for 14 days. All the intervention was given by the same person. Blank control group and model control group were as follows: rats were immobilized in the same restrainer for 20 min without acupuncture intervention.

### 2.5. Samples Obtaining

After the measurement of caudal artery blood pressure on the 15th day, rats were sacrificed after being anesthetized by intraperitoneal injection of 100 g/L chloral hydrate (500 mg/kg). Hippocampus were obtained on the ice, washed with 4°C saline, dried, fixed in liquid nitrogen, and stored at −80°C refrigerator.

### 2.6. Measurement of Blood Pressure

Two hours after the stimulation of model preparation, systolic pressure of caudal artery was measured in a controlled environment at of a temperature of (22 ± 2)°C. Sober rats were preheated in 36°C for 15 minutes. Then the systolic pressure was measured with noninvasive blood pressure instrument when the rat is quiet and conscious. Each rat was measured for 3 times with the average blood pressure recorded. Blood pressure was recorded one day before and on the 3rd, 5th, 7th, 9th, 11th, 13th, and 15th day after modeling.

### 2.7. Sample Preparation and Microarray Image Analysis

Total RNA was separately extracted from all the 27 individual samples using the mirVana miRNA isolation kit (P/N AM1552, Life Technologies, USA) under the manufacturer's instructions. The quality of the extracted total RNA was measured by the NanoDropND-1000 (Thermo Scientific, USA) and RNA integrity was assessed by Agilent 2100 bioanalysis (Agilent Technologies, USA).

Microarray analysis was performed and repeated 3 times using a biological sample in each group with Gene Chip Rat Gene 2.0 ST Array (Affymetrix, USA) containing 610400 distinct probes corresponding to 16771 well-annotated genes. Total RNA of each sample was used for labeling and array hybridization in the following steps. (1) Prepare the poly-A RNA controls. (2) Synthesize first-strand cDNA. (3) Synthesize second-strand cDNA. (4) Synthesize cRNA by in vitro transcription. (5) Synthesize 2nd-cycle cDNA. (6) Hydrolyze using RNase H. (7) Fragment and label the single-stranded cDNA. (8) Array hybridization using the Affymetrix Gene Chip 645 System followed by washing with the Affymetrix Gene Chip 450 System. (9) Array scanning using the Affymetrix Gene Chip 7G microarray scanner (Affymetrix, USA). The labeling and hybridization steps were carried out according to the Affymetrix protocol (Gene Chip WT PLUS Reagent Kit, P/N 902280, Affymetrix, USA).

### 2.8. Data Analysis

Scanned images (CEL format) were then imported into Affymetrix Expression Console software for grid alignment and expression data analysis. Expression data were normalized through quantile normalization and the Robust Multichip Average (RMA+SKETCH) algorithm included in the Affymetrix Expression Console software (Affymetrix, USA). The CHP files (Affymetrix, USA) were generated after normalization. The 27 gene level files were imported into Transcriptome Analysis Console further analysis. Gene expression profiles of model control group were compared to those from blank control group or model + EA group. The changed genes were selected by the following criteria: *P* < 0.05 and fold changes ≥ 1.5.

### 2.9. Gene Functional Annotation

The DAVID 6.7b of DAVID Bioinformatics Resources (http://david.abcc.ncifcrf.gov/) was used to perform gene ontology (GO) enrichment analysis. The biological process, cellular component, and molecular functions were analysed simultaneously. Differentially expressed genes of model control group (versus blank control group) or model + EA group (versus model control group) dataset were selected and were tested against the background set of all genes present in the Affymetrix GeneChip Gene 2.0 ST Array.

### 2.10. KEGG Pathway Analysis

Similarly, pathway analysis was used to determine the significant pathway of the differential genes according to Kyoto Encyclopedia of Genes and Genomes (KEGG) (http://www.genome.jp/kegg/). We used Fisher's exact test to select the significant pathway, and the threshold of significance was defined by the *P* value and FDR.

### 2.11. Clustering and Tree View for Identified Transcripts

Hierarchical clustering was performed with help of Cluster 3.0 (Michael Eisen, USA). A list was prepared of the selected genes to be clustered. The normal signal data of selected genes were adjusted to log transform data. Then the data was arranged according to the requirements of Cluster 3.0 and “median” was selected for center genes and arrays. After that, the results of clustering of selected genes were presented by Java Tree View and exported to the images.

### 2.12. Selected Differentially Expressed Genes Were Validated Using qRT-PCR

Total RNA was separately extracted from the 27 individual samples using the mirVana miRNA isolation kit (P/N AM1552, Life Technologies, USA) in accordance with the manufacturer's instructions. Total RNA was reverse-transcribed to cDNA with a reverse transcription kit (transcriptor first-strand cDNA Synthesis Kit, Life Technologies, USA) under the manufacturer's instructions. The reaction was performed using the following program, 25°C for 10 min, 55°C for 30 min, and 85°C for 5 min, and then stored at 4°C environment. Quantitative RT-PCR method was used to detect gene expression by real-time PCR instrument (Applied Biosystems, USA, 7900HT real-time system) with Power SYBR Green PCR Master Mix kit (Applied Biosystems, 4367659). According to GenBank sequences, we used primer express software (Applied Biosystems, USA) to design primers (Table 1). *β*-actin was selected as an internal reference. Amplification conditions are as follows: 1 cycle of 10 min at 95°C, 40 cycles of 15 sec at 95°C, and 1 min at 60°C. After cycling, a melting protocol was performed with 15 sec at 95°C, 15 sec at 60°C, and 15 sec at 95°C in the end. After the end of the experiment RQ Manager 1.2.1 (Applied Biosystems, USA) and Data Assist V3.0 software (Applied Biosystems, USA) were used for the calculation of Ct value. ΔΔCT method was used for the relative quantification of the gene expression; target gene is =2^−ΔΔCT^.

## 3. Results

### 3.1. Blood Pressure

Systolic blood pressure of Wistar rats in blank control group stayed normal through the whole process of the experiment. Systolic blood pressure in model control group reached higher than 120 mm Hg (*P* < 0.01) on the 3rd day and kept increasing to a significantly higher level when measured on the 3rd, 5th, 7th, 9th, 11th, 13th, and 15th days during model preparation (*P* < 0.01), compared with that of the blank control group. In the modeling process, model control group was of elevated blood pressure, irritation, squealing, gnawing, hard stool, yellow urination, rough hair, and bloodshot eyes. With the value kept in the range between 120 and 139 mm Hg, the model was successfully established.

**Figure 1:**
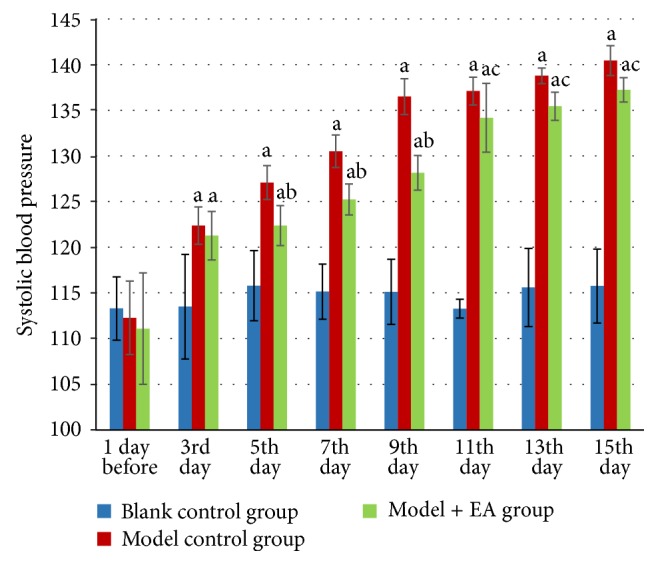
Effects of EA on systolic blood pressure of stress-induced prehypertension rats. Note: a means *P* < 0.01, versus blank control group; b means *P* < 0.01 and c means *P* < 0.05 versus model control group. *N* = 9 animals per group.

Compared with model control group, systolic blood pressure in model + EA group remarkably decreased on the 5th, 7th, and 9th day (*P* < 0.01) and 11th, 13th, and 15th days during EA intervention (*P* < 0.05), indicating that EA at Taichong (LR3) and Quchi (LI11) can significantly lower the blood pressure of stress-induced prehypertension rats with better short-term effects (Figure 1).

### 3.2. Gene Expression Changes

Gene microarray analysis showed that there were 307 gene expressions changed in model control group compared to blank control group; *P* < 0.05 and fold change ≥ 1.5 were identified between these two groups. Of these, 73 were downregulated and 234 were upregulated. EA intervention changed 383 gene expressions compared with model control group; *P* < 0.05 and fold change ≥ 1.5 were identified between model control group and model + EA group. Among these genes, 273 were downregulated and 110 were upregulated.

Gene microarray analysis showed that there were 98 genes whose expression was upregulated in the model control group (compared with blank control group) but was downregulated in model + EA group (compared with model control group) (Table 2) and 39 genes whose expression was downregulated in the model control group (compared with blank control group) but upregulated in model + EA group (compared with model control group) (Table 3).

### 3.3. GO Analysis

**Figure 2:**
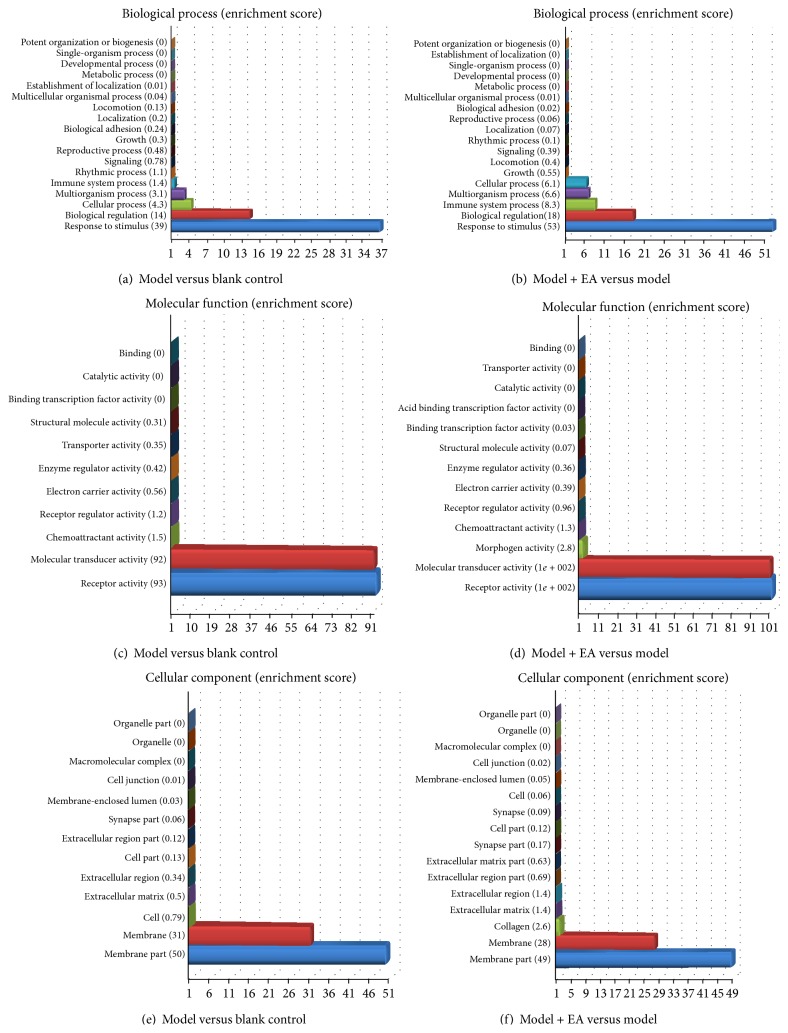
Enriched gene ontology (GO) terms according to biological processes, molecular functions, and cellular components. GO terms are ordered by enrichment score with highest enriched term at the bottom of the list. Differentially expressed transcripts involved in the term (count) with *P* < 0.05 and fold change > 1.5 were included.

To identify the biological processes associated with gene expression changes with EA treatment, we used the DAVID 6.7b of DAVID Bioinformatics Resources (http://david.abcc.ncifcrf.gov/) to perform gene ontology (GO) enrichment analysis. Enriched GO terms are displayed in Figure 2 and are arranged according to biological processes, molecular functions, and cellular components. The categories that were significantly enriched in our gene set were response to stimulus, biological regulation, cellular process, immune system process, multiorganism process, membrane part, receptor activity and molecular transducer activity, and so on.

### 3.4. Pathway Analysis

Further functional pathway analysis showed that, compared with blank control group, changed genes in model control group were involved in 26 KEGG pathways (*P* < 0.05) (Table 4). Compared with model control group, changed genes in model + EA group were mainly related to 23 KEGG pathways (Table 5).

### 3.5. Validation of Expression of Genes by Cluster Analysis and qPCR

**Figure 3:**
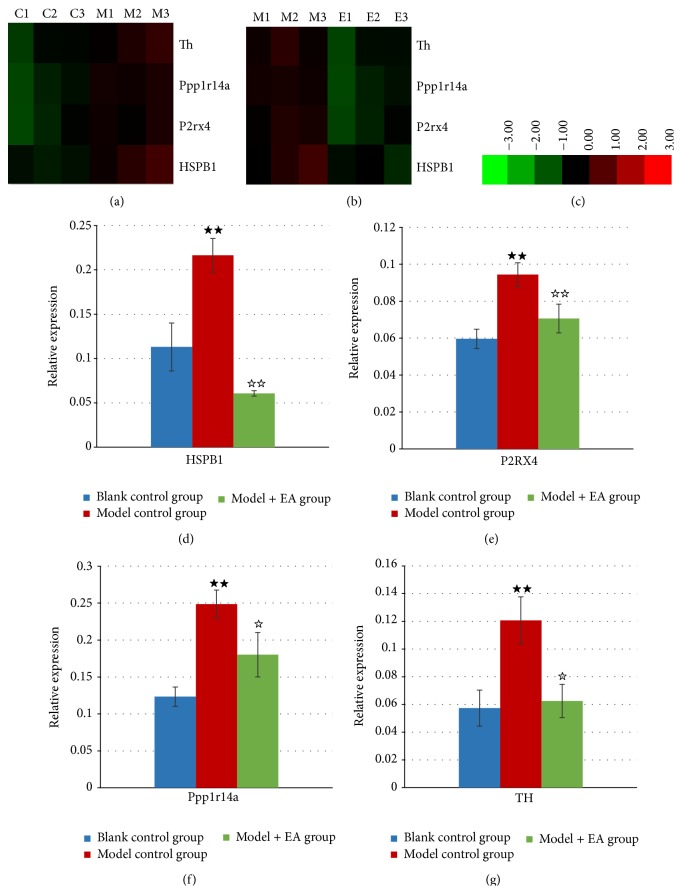
Selected transcripts clustered and validated with real-time PCR. After pathway analysis, we selected genes involved in major pathways (the common pathways in Tables 3 and 4). First, selected genes were clustered using Cluster 3.0 according to the LOG value ((a)–(c)). Red is relatively upregulated and green is relatively downregulated in different samples. The three “Cs” are the three blank control samples. The three “Ms” are the model control samples. The three “Es” are the EA + model samples. Then, to verify the reliability of the microarray analysis, we verified these selected genes from the clustering diagram using quantitative real-time RT-PCR. Gene expressions in blank control group, model control group, and EA + model group were presented in ((d)–(g)). These transcripts analyzed here showed coherent profiles with cluster ((a)–(c)). Note: ^★★^ *P* < 0.01, versus blank control group; ^☆^ *P* < 0.05 and ^☆☆^ *P* < 0.01, versus model control group.

To validate the microarray results, we selected several transcripts to cluster (Figure 3(a)) and validated the clustering result using quantitative real-time PCR (Figure 3(b)). These genes were selected based on fold change differences, previous association with blood pressure regulation, and/or involvement in processes or pathways that may influence blood pressure. The expression of P2RX4 (*P* < 0.01), Ppp1r14a (*P* < 0.01), HSPB1 (*P* < 0.01), and TH (*P* < 0.01) was significantly higher in model control group compared to blank control group. P2RX4 (*P* = 0.018), Ppp1r14a (*P* = 0.04), HSPB1 (*P* < 0.01), and TH (*P* < 0.01) expression were significantly lower in model + EA group when compared to model control group. To some extent, the identified results confirmed the reliability of the array analysis.

## 4. Discussion

Stress-induced hypertension caused by the chronic and excessive psychosocial stress is crucial for the prevalence of coronary diseases [2]. Present studies showed that the disorders of hypothalamus-pituitary-adrenal cortex (HPA) axis [3], sympathetic nerve-adrenal medulla (SAM) [4] system, and renin-angiotensin-aldosterone system (RAS) [5] would lead to stress-induced hypertension. And it has already been proved that adults in the prehypertension group were more likely to progress to clinical hypertension and that there was a significant association with the risk of stroke and cardiovascular diseases (CVDs) [17]. So it is important to pay attention to the prevention and treatment of prehypertension to reduce the risk of CVDs. However, many researches concentrated on antihypertensive treatment in the middle and late stages; fewer researches focused on intervention of prehypertension. Therefore, in this study, we focused on the early stage of prehypertension to observe EA's antihypertensive effects.

In the present study, hypertension was induced by the stress of electric foot-shocks combined with generated noise [6, 18]. We also observed that, after the first 2-3 days of stress, rats in the model control group had obviously changes in performance and behavior, including defensiveness and restlessness. Our results show that, compared with the blank control group, rats in the model control group got significantly increased blood pressure from the third day (systolic blood pressure was higher than 120 mm Hg), suggesting a successfully prepared prehypertension model. The blood pressure continuously rose and reached higher than 140 mm Hg on the 15th day. Therefore, the rats in the model control group experienced a significant increase of systolic blood pressure after exposure to stress, supporting the theory that repeated chronic physical or emotional stress could lead to the elevation of blood pressure.

In Traditional Chinese Medicine (TCM), syndrome (also known as “pattern” or “zheng”) is the basic unit and a key concept; all diagnostic and therapeutic methods in TCM are based on the differentiation of TCM syndrome [19]. Previous studies have shown that syndromes of hypertension are always divided into excess-syndrome and deficiency-syndrome, among which the liver yang hyperactivity type hypertension is the most common clinical syndrome [20]. Studies demonstrated that symptoms like dizziness, headache, anxiety, and irritability that belong to liver qi stagnation and liver yang hyperactivity occurred in the prehypertension stage [21]. In the modeling process of our study, stress-induced hypertension rats were of elevated blood pressure, irritation, squealing, gnawing, hard stool, yellow urination, rough hair, and bloodshot eyes, which were similar to the performance of liver yang hyperactivity syndrome of human with hypertension. Judging from the theory of “preventive treatment of disease,” TCM, especially the acupuncture, shows its unique advantages in preventing and treating prehypertension and high blood pressure [13, 14]. In TCM, the acupuncture could reduce blood pressure by unblocking the meridians and pacifying the liver to subdue yang. In TCM theory,* Quchi* (LI11, He-sea point of large intestine meridian) and* Taichong* (LR3, Yuan-source point of liver meridian) can treat hypertension with liver yang hyperactivity syndrome by pacifying the liver to subdue yang and promoting qi circulation to resolve depression. Modern studies also confirmed that electroacupuncture at* Quchi* (LI11) and* Taichong* (LR3) can remarkably reduce blood pressure [22]. Our study showed that, after EA treatment, compared with the model control group, the blood pressure of model + EA group decreased significantly from the fifth day, suggesting EA can reduce blood pressure of stress-induced prehypertension rats, which was consistent with previous studies.

Gene chip, which detects and analyzes lots of nucleic acid sequences simultaneously with fast speed and high degree of automation, is a new method for the scanning of multiple gene expressions. Therefore, it has been widely applied in medical diagnosis and pathology analysis [23]. Hypertension is a polygenic disease that is related to the interaction of genetic and environmental factors. Gene chip technology, characterized by high sensitivity and throughput, provides a platform for the study of multiple genes influencing hypertension [24]. Therefore, the scanning of all the related gene expression and the analysis of the differences between groups could study the regulatory mechanism of hypertension in a more systematic way. However, fewer researches are focused on the gene expression in prehypertension and the relation between prehypertension and gene expression has not been confirmed. In this study, gene chip results showed that significant differences between gene expressions existed among three groups. Altered genes include HSPB1 that is related to the contraction of vascular smooth muscle; Ppp1r14a and P2RX4 that are related to the regulation of Ca^2+^ concentration; and TH which is related to the sympathetic nerve excitability.

It is proved that hypertension is preceded by the damage of target organs. Besides, the abnormal vascular function, which may be the reason for the damage of target organs like heart, brain, and kidneys, is an important initial link in the development of cardiovascular and cerebrovascular diseases. It is also demonstrated that impaired vascular function is closely associated with inflammation and the level of oxidative stress [9]. Gene chip results in our study showed that HSPB1, which is related to the cytoskeleton and the contraction of vascular smooth muscle, gets upregulated in the model control group when compared with the blank control group but downregulated in model + EA group when compared with the model control group.

Heat shock protein beta-1 (HSPB1), also known as heat shock protein 25 (HSP25), encodes HSP25 protein. HSPB1 belongs to the heat shock protein (HSP) family, which plays an important role in maintaining the stability of the body. HSP genes were activated when the cell was damaged by stressors of various kinds and therefore acted as a biomarker of stress response of the cell. Activated HSP genes can improve cell tolerance to stressors, take part in the endogenous protective mechanisms of cardiovascular system, and play an important role in the pathological process of cardiovascular diseases [25]. It has been reported that HSPB1 is the substrate of MAPK signal molecules and is involved in the regulation of the actin. In stress conditions like ischemia, HSPB1 can enhance myocardial cells' resistance to ischemic injury [26], indicating that HSPB1 is an important kind of heat shock protein with protective function in myocardial tissues. The mechanism may concern the stabilization and repair of the structure of damaged cytoskeletal proteins or nuclear proteins of myocardial tissue.

The gene chip results showed that, after model preparation, the expression of HSPB1 (FC = 1.88297) gets upregulated in the model control group when compared with the blank control group. The elevated expression of HSPB1 gene indicates that endogenous protection of the cardiovascular system has been activated by stress. This result also proved that HSPB1 is involved in the development of hypertension, which is consistent with previous researches. After EA intervention, compared with the model control group, the expression of HSPB1 (FC = −2.52067) remarkably decreased in model + EA group. PCR result is consistent with that of gene chip test. Therefore, we speculate that EA could improve the stress state of the rat and slow down the cellular stress responses to avoid the excessive stress damage while lowering blood pressure. Its specific mechanism needs to be further studied.

Calcium ion (Ca^2+^) plays a major role in the pathogenesis of primary hypertension. It can influence the electrical activity and contraction of myocardial cell and vascular smooth muscle cell. The occurrence of hypertension is closely related to Ca^2+^ transport defect. Studies found that hypertension patients were of abnormal Ca^2+^ metabolism, especially the excessive accumulation of intracellular Ca^2+^ and dysfunction of cell membrane ion transport system [27, 28]. It was demonstrated that needle stimulation at deep peroneal nerve could lower the blood pressure of rats with stress-induced hypertension. The possible explanation of the mechanism may be the influx of extracellular Ca^2+^ in the centre or the regulation of calmodulin and calcium/calmodulin-dependent protein kinase-II. Our gene chip results showed that, after model preparation by electric foot-shocks combined with generated noise, the expression of protein phosphatase 1 regulatory subunit 14A (Ppp1r14a) (FC = 1.66752) and P2X purinoceptor 4 (P2RX4) (FC = 1.52805) which are related to Ca^2+^ signal transduction was upregulated in the model control group when compared with the blank control group.

Protein phosphatase 1 regulatory subunit 14A (PPP1R14A), also known as CPI-17, is mainly expressed in vascular and visceral smooth muscle cells. It is an important molecule that regulates calcium sensitivity. It can enhance the contraction of smooth muscle cells, with the function regulated by various kinds of protein kinases and phosphatases [29]. The present study shows that PKC/CPI-17 signaling pathway is an important link for the occurrence of hypertension [30]. P2X4 receptor, encoded by P2rx4 gene, is a ligand-gated ion channel receptor generating inward current when activated. It belongs to P2X receptor family whose ligand is adenosine triphosphate (ATP). When activated by ATP and its homologue, P2X4 can significantly increase intracellular Ca^2+^ concentration and transmit the signal to the downstream signaling molecules to reduce NO content. Therefore, it is crucial for elevating blood pressure and reconstructing blood vessel. According to gene chip results, after EA intervention, the expression of Ppp1r14a (FC = −1.6519) and P2RX4 (FC = −1.5014) remarkably decreased, compared with the model control group. PCR result is consistent with that of gene chip test. Therefore, Ppp1r14a and P2RX4 genes may be involved in the pathogenesis of stress-induced prehypertension. EA can lower the blood pressure, possibly by regulating Ppp1r14a and P2RX4 gene to correct the Ca^2+^ concentration and improve the contractile function of vascular smooth muscle.

Many studies have shown that the pathogenesis of stress-induced hypertension is associated with overactivity of sympathetic nervous system. The intense and acute stress will lead to hyperactivity of hypothalamic pituitary adrenal (HPA) axis, affecting the function of sympathetic nerve and endocrine system, and thereby causing the overactivity of sympathetic nerve, leading to increased contraction of small resistance arteries and the elevation of blood pressure [31]. Tyrosine hydroxylase (TH), the rate limiting enzyme in the synthesis of catecholamine, is a specific marker of sympathetic nerve. It plays an important role in regulating central and sympathetic nervous systems and cardiovascular functions [32]. Current concept regards overexpression of mRNA and protein of TH gene triggered by TH gene amplification as the possible molecular biological mechanism of hypertension with hyperactivity of liver yang syndrome since increased TH activity raises plasma concentration of norepinephrine (NE) and epinephrine (E) [33]. It is further speculated that TH gene may be a candidate gene for the etiology of hypertension and is crucial for the pathogenesis of hypertension of human [34]. The gene chip results showed that, after modeling by electric foot-shocks combined with generated noise, the expression of TH (FC = 1.93235) in the hypothalamus of the model control group was significantly upregulated compared with the blank control group. This result is consistent with Tao's research reports [35]. Therefore, the expression of TH gene already elevates in the prophase of stress-induced hypertension, with a risen blood pressure as the result of overactivity of TH. After EA intervention, compared with model control group, expression of TH gene (FC = −1.98873) was significantly downregulated in model + EA group. PCR result is consistent with that of gene chip test. Therefore, we proved that EA at* Taichong* (LR3) and* Quchi* (LI11) can reduce the increased activity of hypothalamic TH caused by stress.

In general, this is the study to give a systematic explanation of EA at* Taichong* (LR3) and* Quchi*'s (LI11) antihypertensive effect on stress-induced hypertension from the aspect of gene. Our result will provide basis for the further research of molecular pathogenesis of stress-induced prehypertension and the mechanism of EA's influence on it. However, the physiological and pathological mechanism of and the interaction between these differentially expressed genes still could not be fully explained. The potential mechanism remains to be determined.

## 5. Conclusions

In general, our study proved that EA at* Taichong* (LR3) and* Quchi* (LI11) can significantly lower the blood pressure of stress-induced prehypertension rats and affect its gene expression profile in hypothalamus. It may perform antihypertensive regulation on stress-induced prehypertension via various ways. We speculate that genes and their signal transduction pathways related to the contraction of vascular smooth muscle, the regulation of concentration of Ca^2+^, and excitability of sympathetic nerve may be involved in the mechanism. There may be a network formed by the interrelation, interaction, and intercross of multiple signal pathways that jointly have effect on stress-induced prehypertension.

## Figures and Tables

**Table 1:** Sequences of primers employed for RT-PCR and their anticipated PCR product size.

Gene	Primer	Oligonucleotide sequences 5′-3′	Length (bp)
GADPH	Front	5′-CAGGGCTGCCTTCTCTTGTG-3′	217
	Rear	5′-ACCAGCATCACCCCATTTGA-3′	

P2rx4	Front	5′-ACCAACACCTCTCAGCTTGG-3′	124
	Rear	5′-TGCTCTGTGTCTGGTTCACG-3′	

Ppp1r14a	Front	5′-GCGAGTCACCGTCAAATACG-3′	120
	Rear	5′-ACCTCATCTGGCATGTCTGC-3′	

Hspb1	Front	5′-CGGTGCTTCACCCGGAAATA-3′	152
	Rear	5′-TCGAAAGTGACCGGAATGGT-3′	

Th	Front	5′-GTCACGTCCCCAAGGTTCAT-3′	159
	Rear	5′-AGCATTCCCATCCCTCTCCT-3′	

**Table 2:** Genes upregulated in model control group (compared with blank control group) but downregulated in model + EA group (compared with model control group).

Gene abbr.	FC_1_	*P*	FC_2_	*P*
Vom2r38	1.58211	0.0115145	−2.21593	0.000322224
Vom1r82	1.65924	0.0517768	−2.14824	0.00757208
Spetex-2G	3.37346	0.0234389	−2.8499	0.0441933
Rpl30	2.9497	0.011107	−3.23517	0.00708636
P2rx4	1.52805	0.00226395	−1.5014	0.00388464
Olr987	4.75787	0.000407988	−3.20186	0.00310371
Oaz2	3.22396	0.0286101	−3.24048	0.028068
LOC689635	1.93938	0.0351156	−2.21265	0.015294
LOC688318	5.05198	0.00784832	−4.31929	0.0135292
IgG-2a	1.6542	0.0449334	−1.96557	0.0117183
Hspb1	1.67043	0.00021141	−1.54732	0.000712861
Gpd1	1.77399	0.00365871	−1.76389	0.00389163
Fv1	2.03917	0.0110901	−2.10111	0.00888503
Fkbp5	1.56254	0.00049632	−1.56525	0.000482253
Cyp27b1	2.33918	0.0177387	−2.35641	0.0170103
Chordc1	1.53554	0.0000132173	−1.70829	0.0000018127
Zbtb16	1.57806	0.00178346	−1.58084	0.00173813
Vom2r72	1.61423	0.0612813	−1.76362	0.0315944
Vom1r56	1.75781	0.0654545	−2.28946	0.0125168
Vom1r14	3.97844	0.0147139	−4.73732	0.00781451
Spetex-2A	3.13715	0.0338017	−3.13899	0.033729
Th	1.93235	0.0326253	−1.98873	0.0271016
Ppp1r14a	1.66752	0.00267407	−1.6519	0.00300997

*Note*. Abbr.: gene abbreviation. FC_1_: gene expression level in model control group/gene expression level in blank control group. FC_2_: gene expression level in model + EA group/gene expression level in model control group. Positive values indicate higher expression in model samples, while negative values indicate lower expression in model + EA samples.

**Table 3:** Genes downregulated in the model control group (compared with blank control group) but upregulated in model + EA group (compared with model control group).

Gene abbr.	FC_1_	*P*	FC_2_	*P*
Vtn	−1.61202	0.0118045	1.68522	0.00727322
Usp18	−1.53995	0.193412	2.74303	0.00859159
Tnfsf10	−2.08653	0.00773606	2.67158	0.00126058
Tmem176a	−1.57271	0.0049936	1.71754	0.00161366
Slfn5	−1.69337	0.00676306	1.84538	0.00270496
Slco1a2	−1.54868	0.000222761	1.66634	0.0000642471
Slc22a8	−1.76993	0.0306791	2.14172	0.00731394
Serping1	−1.76026	0.0578559	2.28307	0.0107471
RT1-Da	−2.20008	0.0400228	2.17055	0.042878
Ppap2c	−1.55321	0.0003064	1.68218	0.0000819669
Pllp	−1.71886	0.00290608	1.63516	0.00525928
Ms4a4c	−1.71971	0.00686195	1.78826	0.00456226
Ly86	−1.72914	0.0131221	1.76035	0.0110967
LOC688318	−3.18027	0.0123412	4.23053	0.00350328
Klrk1	−1.58334	0.049992	1.67771	0.0309681
Klra1	−2.15395	0.00557922	2.11286	0.0064672
Cxcl13	−4.32406	0.0211367	4.75402	0.0156071
Cga	−3.39698	0.0310596	3.80055	0.0209263
Ccrl1	−1.69678	0.00274726	1.69915	0.00270131
Car4	−1.84764	0.0000503703	1.56076	0.00062746
Bmp4	−1.57763	0.00336173	1.75791	0.000805044

*Note*. Abbr.: gene abbreviation. FC_1_: gene expression level in model control group/gene expression level in blank control group. FC_2_: gene expression level in model + EA group/gene expression level in model control group. Positive values indicate higher expression in model samples, while negative values indicate lower expression in model + EA samples.

**Table 4:** KEGG pathways of altered genes in model control group compared with blank control group.

KEGG pathway	Genes
rno04640: hematopoietic cell lineage	IL3, CD37, CD4, RT1-DA, and CSF1R
rno05310: asthma	IL3, FCER1G, and RT1-DA
rno03010: ribosome	RPL18, RPL30, RGD1563705, and RPL22L1
rno04612: antigen processing and presentation	IFNA2, CD4, RT1-DA, and CD74
rno04650: natural killer cell mediated cytotoxicity	IFNA2, TNFSF10, KLRK1, and FCER1G
rno05320: autoimmune thyroid disease	IFNA2, CGA, and RT1-DA
rno04060: cytokine-cytokine receptor interaction	IL3, IFNA2, TNFSF10, CXCL13, and IL10RA
rno00565: ether lipid metabolism	PPAP2C, PLA2G7
rno05340: primary immunodeficiency	PTPRC, CD4
rno05217: basal cell carcinoma	BMP4, FZD2
rno00564: glycerophospholipid metabolism	GPD1, PPAP2C
rno04630: Jak-STAT signaling pathway	IL3, IFNA2, and IL10RA
rno04664: Fc epsilon RI signaling pathway	IL3, FCER1G
rno04514: cell adhesion molecules (CAMs)	PTPRC, CD4, and RT1-DA
rno04210: apoptosis	IL3, TNFSF10
rno04666: Fc gamma R-mediated phagocytosis	PTPRC, PPAP2C
rno05322: systemic lupus erythematosus	C3, RT1-DA
rno04912: GnRH signaling pathway	CGA, MMP14
rno04660: T cell receptor signaling pathway	PTPRC, CD4
rno04080: neuroactive ligand-receptor interaction	CGA, TAAR9, PTGDRL, and TAAR3
rno05012: Parkinson's disease	SLC6A3, TH
rno05200: pathways in cancer	BMP4, FZD2, ZBTB16, and CSF1R
rno04350: TGF-beta signaling pathway	BMP4
rno04010: MAPK signaling pathway	HSPB1
rno04270: vascular smooth muscle contraction	PPP1R14A
rno04370: VEGF signaling pathway	HSPB1

**Table 5:** KEGG pathways of altered genes in EA + model group compared with model control group.

KEGG pathway	Genes
rno04612: antigen processing and presentation	RT1-CE16, CD4, RT1-DA, and CD74
rno05322: systemic lupus erythematosus	RGD1564953, LOC679994, C3, and RT1-DA
rno05320: autoimmune thyroid disease	CGA, RT1-CE16, and RT1-DA
rno04742: taste transduction	TAS2R109, TAS2R108
rno05217: basal cell carcinoma	BMP4, FZD2
rno05332: graft-versus-host disease	RT1-CE16, RT1-DA
rno05330: allograft rejection	RT1-CE16, RT1-DA
rno04940: type I diabetes mellitus	RT1-CE16, RT1-DA
rno00564: glycerophospholipid metabolism	GPD1, PPAP2C
rno04610: complement and coagulation cascades	C3, SERPING1
rno05012: Parkinson's disease	NDUFS7, LOC691015, and TH
rno04514: cell adhesion molecules (CAMs)	RT1-CE16, CD4, and RT1-DA
rno05416: viral myocarditis	RT1-CE16, RT1-DA
rno00190: oxidative phosphorylation	NDUFS7, LOC691015
rno04080: neuroactive ligand-receptor interaction	CGA, PTGDRL, and TAAR3
rno05016: Huntington's disease	NDUFS7, LOC691015
rno05010: Alzheimer's disease	NDUFS7, LOC691015
rno04060: cytokine-cytokine receptor interaction	TNFSF10, CSF1R
rno04010: MAPK signaling pathway	HSPB1
rno04370: VEGF signaling pathway	HSPB1
rno04350: TGF-beta signaling pathway	BMP4
rno04210: apoptosis	TNFSF10
rno04270: vascular smooth muscle contraction	PPP1R14A
